# Supplementary material for: Factors behind Antibiotic Therapy: A Survey of Primary Care Pediatricians in Lombardy
Source: Int J Environ Res Public Health. 2024 Aug 18;21(8):1091. doi: 10.3390/ijerph21081091 (PMC11354739; doi:10.3390/ijerph21081091)
Supplement: Supplementary file 1 [file ijerph-21-01091-s001.zip › ijerph-3086037 Supplementary Material.pdf]

Le conoscenze su antibioticoterapia e prescrizione di antibiotici in pediatria territoriale

Gentile Collega,

in relazione ad uno delle principali problematiche di Sanità Pubblica dei prossimi anni, l'Università degli Studi di Milano desidera invitarla alla compilazione di un breve questionario riguardante la conoscenza in ambito di antibiotico terapia e dell'impatto in termini di antibiotico-resistenza della non corretta prescrizione antibiotica. I dati raccolti saranno importanti per poter intraprendere iniziative per migliorare le competenze su questa tematica.

Le risposte da Lei fornite saranno raccolte in maniera completamente anonima e unicamente a fini accademici.

Il questionario è rivolto ai pediatri di famiglia e ha una durata di circa 10 minuti.

Grazie per il Suo tempo.

Si ringrazia SICuPP Lombardia per la preziosa collaborazione.

1. **Genere**

☐ Maschio
☐ Femmina

2. **Anno di Laurea**

____

3. **Anno di Specializzazione in Pediatria**

____

4. **Ha più di una Specializzazione?**

☐ Sì
☐ No

5. **Di quali altre specializzazioni è in possesso?**

____

6. **Da quanto svolge l'attività di Pediatra di libera Scelta**

☐ Meno di 1 anno
☐ 1 anno
☐ più di 1 anno ma meno di 5 anni
☐ 5 anni o più ma meno di 10 anni
☐ 10 anni o più

7. **Ha seguito corsi, seminari o congressi sull'Antibiotico Resistenza negli ultimi 3 anni?**

☐ No e non sarei interessato a seguirli
☐ No ma vorrei seguirli
☐ Sì ma solo 1
☐ Sì più di 1

8. **Quali sono, secondo lei, le principali cause dell'Antibiotico Resistenza? (Più risposte possibili)**

☐ Utilizzo di antibiotici in casi non indicati
☐ Posologia scorretta di antibiotici nei casi in cui ne sia indicato l'utilizzo
☐ Co-somministrazione di diverse classi antibiotiche quando non necessario
☐ Utilizzo di antibiotici di seconda e terza linea quando non necessario

9. **In un bambino con sintomatologia influenzale, quando prescriverebbe una * terapia antibiotica?**

☐ Mai
☐ In caso di febbre alta (superiore ai 39°) per più di 3 giorni
☐ In caso di febbre alta (superiore ai 39°) per più di 5 giorni
☐ Su richiesta/insistenza del genitore
☐ Quando penso che il quadro si stia complicando

10. **Nel caso di un bambino con otite media acuta, quale antibiotico prescrive come prima scelta? (scelga tutte le risposte che ritiene corrette)**

☐ Amoxicillina
☐ Amoxicillina+acido clavulanico
☐ Macrolidi
☐ Cefalosporine
☐ Dipende dall'età
☐ Dipende dai fattori rischio
☐ Dipende dal quadro clinico
☐ Non prescrivo antibiotici

11. **Nel caso di un bambino con faringotonsillite da SBEGA, quale antibiotico prescrive come prima scelta?**

- ☐ Amoxicillina
- ☐ Amoxicillina+acido clavulanico
- ☐ Macrolidi
- ☐ Cefalosporine
- ☐ Dipende dall'età
- ☐ Dipende dai fattori rischio
- ☐ Dipende dal quadro clinico
- ☐ Non prescrivo antibiotici

12. **Nel caso di un bambino con bronchite, quale antibiotico prescrive come prima scelta?**

- ☐ Amoxicillina
- ☐ Amoxicillina+acido clavulanico
- ☐ Macrolidi
- ☐ Cefalosporine
- ☐ Dipende dall'età
- ☐ Dipende dai fattori rischio
- ☐ Dipende dal quadro clinico
- ☐ Non prescrivo antibiotici

13. **Nel caso di un bambino con laringite, quale antibiotico prescrive come prima scelta?**

- ☐ Amoxicillina
- ☐ Amoxicillina+acido clavulanico
- ☐ Macrolidi
- ☐ Cefalosporine
- ☐ Dipende dall'età
- ☐ Dipende dai fattori rischio
- ☐ Dipende dal quadro clinico
- ☐ Non prescrivo antibiotici

14. **Qual è la classe di antibiotici per cui ha registrato il maggior numero di insuccessi terapeutici**

- ☐ Penicilline
- ☐ Macrolidi
- ☐ Cefalosporine
- ☐ Aminoglicosidi
- ☐ Fluorochinolonici
- ☐ Glicopeptidi
- ☐ Altro: ______________________________

15. **Mettiti in ordine di importanza quale di questi fattori non legati alla prescrizione antibiotica secondo te è maggior causa di antibiotico resistenza**

	Aumento dei viaggi e degli spostamenti internazionali	Inappropriato utilizzo degli antibiotici in zootecnia e in agricoltura	Inappropriata prescrizione di antibiotici in ambito pediatrico	Inappropriata prescrizione di antibiotici nell'adulto	Inappropriata prescrizione di antibiotici nell'anziano
Prima causa					
Seconda causa					
Terza causa					
Quarta causa					
Quinta causa					

16. **Il fenomeno dell'antibiotico-resistenza nei bambini è un fenomeno?**

Assente 1 2 3 4 5 Estremamente frequente

17. **Quali conseguenze può determinare il fenomeno dell'Antibiotico-resistenza (AMR, Antimicrobial resistance) nel bambino? (Più risposte possibili)**

☐ Aumento della mortalità nella popolazione pediatrica
☐ Aumento delle possibilità di sviluppo di eventi avversi alla terapia antibiotica nella popolazione pediatrica
☐ Aumento delle possibilità di infezioni più gravi nella popolazione pediatrica Aumento della mortalità nella popolazione adulta
☐ Aumento delle possibilità di sviluppo di eventi avversi alla terapia antibiotica nella popolazione adulta
☐ Aumento delle possibilità di infezioni più gravi nella popolazione adulta

18. **É a conoscenza dell'esistenza di piani Nazionali per contrastare il fenomeno dell'antibiotico-resistenza in ambito pediatrico?**

☐ No
☐ Sì in Italia
☐ Sì ma in Europa (non in Italia)
☐ Sì ma solo in paesi Extra-UE

19. **Potrebbe essere utile un piano nazionale per contrastare il fenomeno dell'antimicrobico resistenza in ambito pediatrico?**

Assolutamente no 1 2 3 4 5 Estremamente necessario

20. **Parteciperebbe ad un corso sull'Antimicrobial stewardship (serie di interventi coordinati, che hanno lo scopo di promuovere l'uso appropriato degli antimicrobici e che**

indirizzano nella scelta ottimale del farmaco, della dose, della durata della terapia e della via di somministrazione)

Assolutamente no 1 2 3 4 5 6 Ne sarei molto interessato

21. **Quanto l'incertezza diagnostica pensa influenzi le sue decisioni in ambito di antibiotico terapia pediatrica**

☐ Mai
☐ <25% delle volte
☐ Tra il 25% e il 50% delle volte
☐ Tra il 50% e il 75% delle volte
☐ >75% delle volte Sempre

22. **Quanto le lacune conoscitive pensa influenzino le sue decisioni in ambito di antibiotico terapia pediatrica**

☐ Mai
☐ <25% delle volte
☐ Tra il 25% e il 50% delle volte
☐ Tra il 50% e il 75% delle volte
☐ >75% delle volte Sempre

23. **Quanto le richieste dei genitori pensa influenzino le sue decisioni in ambito di antibiotico terapia pediatrica**

☐ Mai
☐ <25% delle volte
☐ Tra il 25% e il 50% delle volte
☐ Tra il 50% e il 75% delle volte
☐ >75% delle volte Sempre

24. **Quanto il poco tempo da dedicare al follow-up pensa influenzi le sue decisioni in ambito di antibiotico terapia pediatrica**

☐ Mai
☐ <25% delle volte
☐ Tra il 25 e il 50% delle volte
☐ Tra il 50% e il 75% delle volte
☐ >75% delle volte Sempre

25. **In quale fascia d'età prescrive prevalentemente antibiotici?**

☐ <1 anno
☐ tra 1 e 2 anni

☐ tra 3 e 6 anni
☐ tra 7 e 10 anni
☐ tra 11 e 13 anni
☐ >14 anni

26. **In un bambino con un'infezione virale le capita di prescrivere antibiotici per prevenire sovrainfezioni batteriche?**

☐ Mai
☐ <25% delle volte
☐ Tra il 25% e il 50% delle volte
☐ Tra il 50% e il 75% delle volte
☐ >75% delle volte Sempre

27. **Diagnostica un'infezione virale, il genitore insiste ripetutamente perché venga impostata una terapia antibiotica; con che frequenza prescrive un antibiotico in questi casi?**

☐ Mai
☐ <25% dei casi
☐ Tra il 25% e il 50% dei casi
☐ Tra il 50% e il 75% dei casi
☐ >75% dei casi Sempre

28. **Quanto pensa che la paura di una denuncia influenzi il suo operare nella prescrizione antibiotica**

Nessun'influenza 1 2 3 4 5 6 Guida le mie scelte

29. **È mai stata intenta nei suoi confronti una causa per aver non prescritto terapia antibiotica?**

☐ Sì
☐ No

30. **Come questa esperienza ha influito sul suo modo di gestire la terapia di un paziente?**

☐ Prescrivo un po' più frequentemente terapia antibiotica
☐ Prescrivo molto più frequentemente terapia antibiotica

31. **È mai stata intenta nei suoi confronti una causa per aver prescritto terapia antibiotica?**

☐ Sì
☐ No

32. **Come questa esperienza ha influito sul suo modo di gestire la terapia di un paziente?**

- ☐ Prescrivo un po' più frequentemente terapia antibiotica
- ☐ Prescrivo molto più frequentemente terapia antibiotica

La ringraziamo per aver dedicato parte del suo tempo per questo studio
